# ﻿Taxonomic study of a novel terrestrial alga, *Spongiosarcinopsisqinghaiensis* sp. nov. (Protosiphonaceae, Chlorophyta), from the Qinghai-Tibet Plateau

**DOI:** 10.3897/phytokeys.204.84886

**Published:** 2022-08-11

**Authors:** Qiufeng Yan, Huan Zhu, Jiao Fang, Benwen Liu, Guoxiang Liu

**Affiliations:** 1 Key Laboratory of Algal Biology, Institute of Hydrobiology, Chinese Academy of Sciences, Wuhan 430072, China Institute of Hydrobiology, Chinese Academy of Sciences Wuhan China; 2 University of Chinese Academy of Sciences, Beijing 100049, China University of Chinese Academy of Sciences Beijing China

**Keywords:** ITS2 secondary structure, new species, phylogenetic analysis, *Spongiosarcinopsis*

## Abstract

There is only one species of *Spongiosarcinopsis* in the literature currently. It was found in gray soil in Russia for the first time. According to molecular data analysis results, the isolated algal strain is most closely related to *Spongiosarcinopsisterrestris*. Unlike *Spongiosarcinopsisterrestris*, the isolated strain was found on soil surfaces at high altitudes, the young vegetative cell is spherical, vegetative cells are relatively large, and pyrenoids are generally fewer. In view of such morphological differences, phylogenetic analysis results, and comparison of ITS2 secondary structure and ultrastructure, the strain isolated in the present study was proposed to be a novel species.

## ﻿Introduction

Algae exist in almost all terrestrial environments on earth, and always appear on and below the soil surface (Metting 1981). Since the end of the 19^th^ century, algae have been recognized as an integral part of the soil microbial community (Starks et al. 1981). Algae that grow on or under soil play important roles in agro-ecosystems and are indicators of soil quality (Trofim et al. 2013). Soil algae have a positive effect on soil formation and stabilization, increasing soil nitrogen content through nitrogen fixation (Metting 1981; Goyal 1997; Kuzyakhmetov 1998). The soil environment is highly heterogeneous, which provides diverse habitats for exploitation by microorganisms, including algae (Trevors and Wellington 1997). Many algae are found in soil habitats, including *Spongiosarcinopsis* (Temraleeva et al. 2018; Temraleeva 2019), *Chlorococcum* (Nostoch 1842), *Hormidium* (Kützing 1843), *Tetracystis* (Brown and Bold 1964), *Chlorosarcinopsis* (Herndon 1958), *Protococcus* (Agardh 1824), *Neochloris* (Starr 1955), and *Sphaerocystis* (Chodat 1897). In view of the diversity and importance of soil green algae, it is necessary to classify and identify them accurately.

*Spongiosarcinopsis* was proposed in 2018, due to its unique morphological characteristics (chloroplasts are spongy, which distinguish it from other genera in the family) and molecular sequence, it is classified within Protosiphonaceae (Temraleeva et al. 2018; Temraleeva 2019). Protosiphonales are divided into four groups, out of which three groups include Stephanosphaeraceae, Chlorococcaceae, and Protosiphonaceae. Protoiphonaceae (Chlorophyta, Chlorophyceae) are characterized by naked zoospores, one to several pyrenoids and a starch envelope. In addition, most algae in the family can produce excessive secondary carotenoids, and most of the algae turn orange (Temraleeva et al. 2017). Based on a literature review, recent studies rarely rely exclusively on morphology when classifying species. This is because many algae exhibit large intraspecific morphological variability, genetically controlled polymorphisms, or environment-induced plasticity (Lurling 2003; Logares et al. 2007). Consequently, more comprehensive taxonomic evidence is required to support the conclusions of taxonomists, including 18S ribosomal DNA (rDNA) and internal transcribed spacer (ITS) rRNA molecular markers, which are commonly used in green algae, in addition to compensatory base pair changes (CBCS), for species differentiation (Leliaert et al. 2014).

The aim of the present study was to combine morphological and molecular phylogenetic analyses of 18S rDNA and ITS rRNA sequences, and compare ITS2 secondary structure and ultrastructure between the two species in the genus *Spongiosarcinopsis*. A novel *Spongiosarcinopsis* sp. was proposed.

## ﻿Materials and methods

### ﻿Isolation and culture of algal strains

Collection of algal strains from the surface of loose sandy soil near Qinghai Lake in September 2020 (37°02'N, 100°44'E, altitude: 3201.8 m). Take an appropriate amount of soil sample into a 2 ml centrifuge tube, add an appropriate amount of distilled water, mix well with a vortex shaker, and inoculate an appropriate amount of suspension with a pipette tip on BG-11 solid medium containing 1.3% agar, and then use a triangular glass rod coated solid slab (Allen 1968), and then incubated at a constant temperature of 25 °C under a 12/12-h light/dark cycle until visible colonies appeared. Then, the algal colonies were transferred to fresh medium until a pure single algal culture was obtained. Transfer the single algal culture to a 96-well plate containing BG-11 liquid medium. The algal strains were stored in the freshwater algae culture bank (Freshwater Algae Culture Collection at the Institute of Hydrobiology) of Institute of aquatic biology, Chinese Academy of Sciences, China(No. 7, Donghu South Road, Wuhan, Hubei), under the Accession No. FACHB-(3451). Morphological observations of native and cultured algal strains were performed using a Leica DM5000B microscope, and photomicrographs were obtained using a Leica DFC320 digital camera.

### ﻿DNA extraction, PCR amplification, sequencing

The pure unialgal cells were disrupted using a beader (3110BX, Biospec Products, Buttersville, USA) with mini beads. The total DNA was extracted using HP Plant DNA Kit (Omega Bio-Tek, GA, USA). PCR amplification was performed using 6 μL of template DNA, 1 μL of each primer, and 42 μL of Master Mix in a 50-μL reaction volume. The 18S rDNA sequences were amplified using the primers 18SR and 18SF (Medlin et al. 1988), and the amplification conditions were as follows: 94 °C for 5 min, followed by 94 °C for 50 s, 55 °C for 50 s, 72 °C for 90 s, and a final extension at 72 °C for 10 min. The primers used to amplify the ITS sequence were NS7m and LR1850 (Bhattacharya et al. 1996), and the amplification conditions were as follows: 32 cycles of 94 °C for 5 min, 94 °C for 1 min, 55 °C for 1 min, 72 °C for 2 min, and a final extension at 72 °Cor 10 min. The PCR products were sequenced by TSINGKE Biotechnologies (China), and the sequences have been deposited in GenBank (http://www.ncbi.nlm.nih.gov/) with login numbers OM333733 and OM333734.

### ﻿Molecular phylogenetic analyses

Based on BLAST search selection as well as possible phylogenetic relationships and broader green algae, 45 18S rDNA and 19 ITS sequences were downloaded from GenBank, and preliminarily aligned using MAFFT 7.3 (Standley 2013). Manual optimization via Seaview (v.5.0) (Gouy et al. 2021). Pairwise distances were plotted against model-corrected distances using MEGA (v.11.0)(Tamura et al. 2021) to evaluate mutational saturation of the alignments saturation in the variable positions, and neither transversion nor transformation reached saturation. Phylogenies were estimated using maximum likelihood (ML) in PhyloSuite (v.1.2.1) (Zhang et al. 2020) and Bayesian inference in MrBayes (v.3.2.2) (Mishra and Thines 2014). The best-fit evolutionary model was selected using hiearchial likelihood ratio tests and Akaike information criterion through Modeltest-NG (Darriba et al. 2020). K2+G+I and K2+G were found to be the best-fit models for 18S rDNA and ITS, respectively. For ML analysis, tree search was realized using a heuristic search option with random addition of sequences (10 replicates) and tree bisection and reconnection branch-swapping algorithm. Statistical reliability was estimated by Bootstrap analysis with 1000 replicates of the dataset for ML. Four Markov chains (three heated chains, one cold chain) were run for Bayesian Markov Chain Monte Carlo analysis for 20 million generations with tree sampling performed for every 10,000 generations. Stationary distribution was assumed when the average standard deviation of split frequencies between the two runs was lower than 0.01. The first 25% of the trees was discarded, the consensus tree with the remaining samples was constructed, and a posteriori probability was inferred. The phylogenetic trees were edited using Figtree1.4.3 (http://tree.bio.ed.ac.uk/software/figtree/).

### ﻿Comparison of ITS2 secondary structure

Implemented the prediction of ITS2 secondary structure in the ITS2 database (http://its2.bioapps.biozentrum.uni-wuerzburg.de/). By comparing the secondary structures of ITS2 of the two algae, the differences in the two structures were analyzed. Cluster W (Larkin et al. 2007) multiple alignment sequence in 4SALE version 1.7 software was used to align ITS2 sequence and secondary structure (Seibel et al. 2008). Secondary structure images edited with Varna v3.1 (Darty et al. 2009).

## ﻿Results

### ﻿ Spongiosarcinopsis qinghaiensis

Taxon classificationPlantaeChlamydomonadalesProtosiphonaceae

Q. YAN & G. LIU sp. nov.

F224EC3C-5E2A-56E8-A6E1-C292EAA59EA2

#### Description.

**Figures 1–6.**
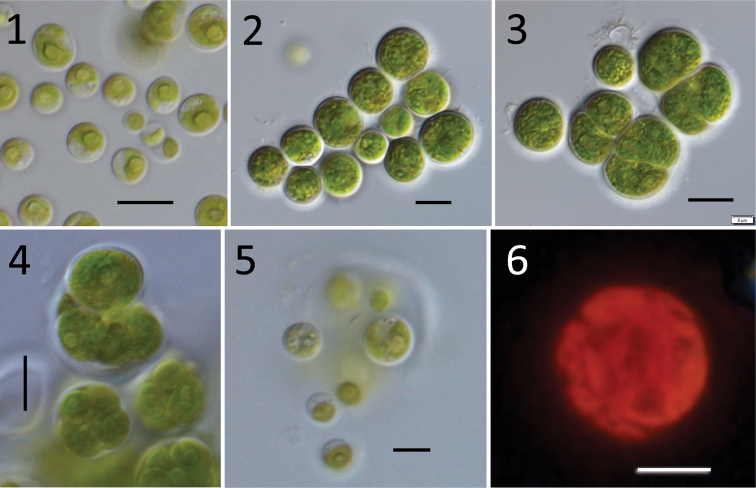
Culture sample morphology of *Spongiosarcinopsisqinghaiensis* sp. nov.: **1** the normal growth state of the algal strain, young cells have a larger protein nucleus **2** mature vegetative cells **3** vegetative cells reproduce through diads **4** aplanosporangia containing aplanospores covered by thin cell walls **5** aplanospores released from aplanosporangia **6** net-like chloroplast under fluorescence. Scale bar: 10 μm (**1–4**); 5 μm (**5–6**).

**Figures 7–10.**
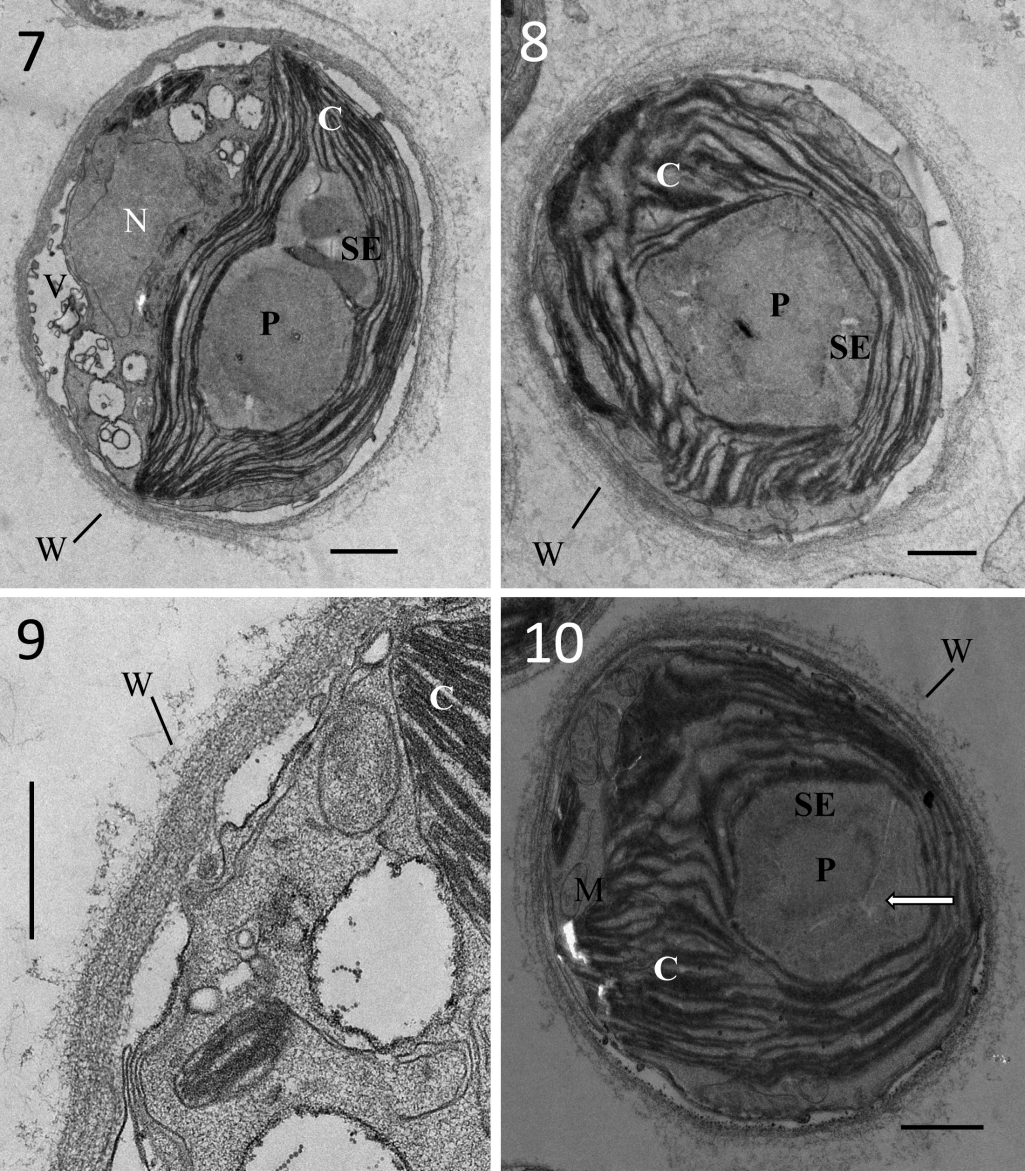
TEM images of *Spongiosarcinopsisqinghaiensis* sp. nov.: **7** cells contain one nucleus, one chloroplast, one pyrenoid covered by starch envelope **8** the starch envelope covering the pyrenoid is not obvious **9** unidentified gelatinous layer outside the cell wall **10** the pyrenoid is covered by segmented starch envelope and penetrated by straight thylakoids. C = chloroplast, N = nucleus, P = pyrenoid, SE = starch envelope of the pyrenoid, W = cell wall. Arrows indicate thylakoids penetrating into the pyrenoid matrix. Scale bar: 1 μm (**7, 8, 10**); 500 nm (**9**).

Found on the soil surface around brackish lakes. Unicellular, spherical (Figs 1, 2), and cell diameter of 6–15 μm. Mature cells often divide into two cells by means of diads (Fig. 3). Cells contain one net-like chloroplast (Fig. 6), lateral, almost filling the entire cell at maturity (Figs 2, 8), one nucleus, and one pyrenoid covered by starch envelope, although not obvious (Figs 7, 8).

The vegetative cell wall is rough and there is an unidentified gelatinous layer outside the cell wall (Fig. 9). The pyrenoid is covered with a segmented starch envelope and penetrated by straight thylakoids (Fig. 10).

Asexual reproduction is achieved through diads or release of aplanospores (Figs 3–5). Aplanosporangia contain eight to 16 spherical aplanospores, and the release process can be observed (Figs 4, 5). No sexual reproduction was observed.

#### Etymology.

The species epithet refers to the Holotype locality (Qinghai Province).

#### Type locality.

Qinghai Lake National Nature Reserve (37°02'N, 100°44'E), Qinghai Province, China; on soil surface.

#### Iconotype.

Fig. 2.

#### Holotype.

QH2015 (HBI), collected by Qiu–Feng Yan and Huan Zhu, 22 September 2020; deposited in the Freshwater Algal Herbarium (HBI), Institute of Hydrobiology, Chinese Academy of Sciences, Wuhan, Hubei Province, China.

#### Distribution.

At present, the algal isolate is only found in China. It grows on loose and moist soil surfaces around brackish lakes.

#### Authentic culture.

Culture strain FACHB-3451 is deposited in the Freshwater Algae Specimen Station, Institute of Hydrobiology, Chinese Academy of Sciences (http://algae.ihb.ac.cn/).

Therefore, *Spongiosarcinopsisqinghaiensis* was found to be different from *Spongiosarcinopsisterrestris*, with respect to young cell shape, habitat, size of vegetative cells, and number of pyrenoids. Registration. http://phycobank.org/103211.

##### ﻿Phylogenetic analyses

The 18S rDNA alignment applied 45 sequences (including 15 Protosiphonaceae sequences), which consisted of 1686 sites, out of which 300 (17.8%) and 208 (12.3%) were variable and parsimony-informative sites, respectively. Nineteen ITS sequences were used for alignment and 546 sites, out of which 327 (59.9%) and 232 (42.5%) were variable and parsimony-informative sites, respectively. Table 1 presents detailed information about the alignment and nucleotide substitution in 18S rDNA and ITS concatenated phylogenies for molecular phylogenetic analysis.

**Table 1.** Detailed information of alignment and nucleotide substitution in 18S and *tuf*A concatenated phylogenetic for ML analysis.

Dataset	18S	ITS
Alignment length	1686	546
Number of sequences	45	19
Parsimony-informative sites	208	232
Invariant sites	1386	219
Best-fit model	K2+G+I	K2+G
Base frequency (A/C/G/T)	0.25/0.21/0.28/0.26	0.25/0.26/0.24/0.25
Saturation test (*Iss/Iss.*c)	0.056<0.836	0.397<0.721

**Figure 11.**
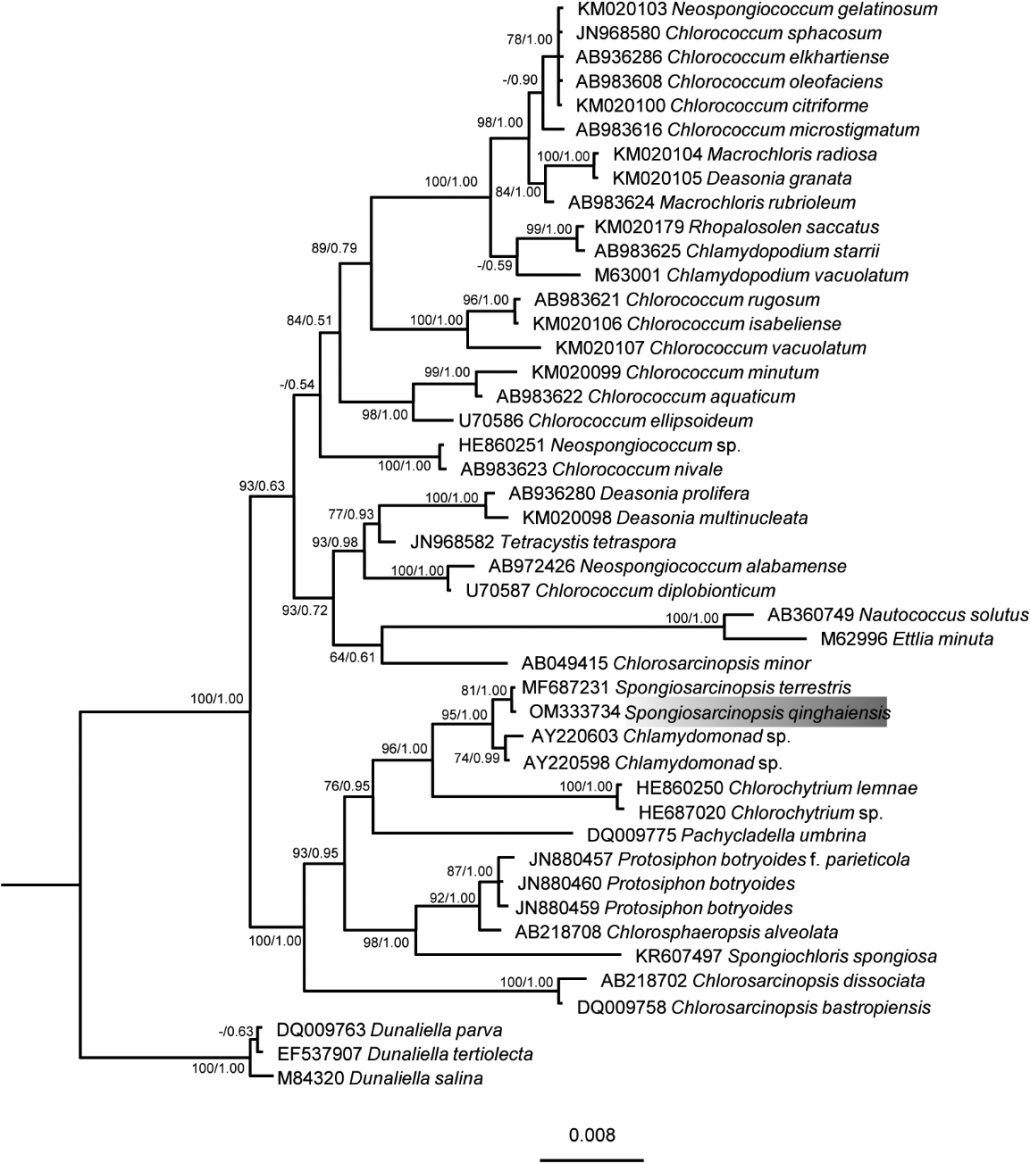
Phylogenetic tree constructed by Bayesian approach based on 18S rDNA sequences. Maximum likelihood bootstrap values and Bayesian posterior probabilities are given on nodes. Values above 0.50 for BI and 50 for ML are given. The gray part shows the new species of this study.

**Figure 12.**
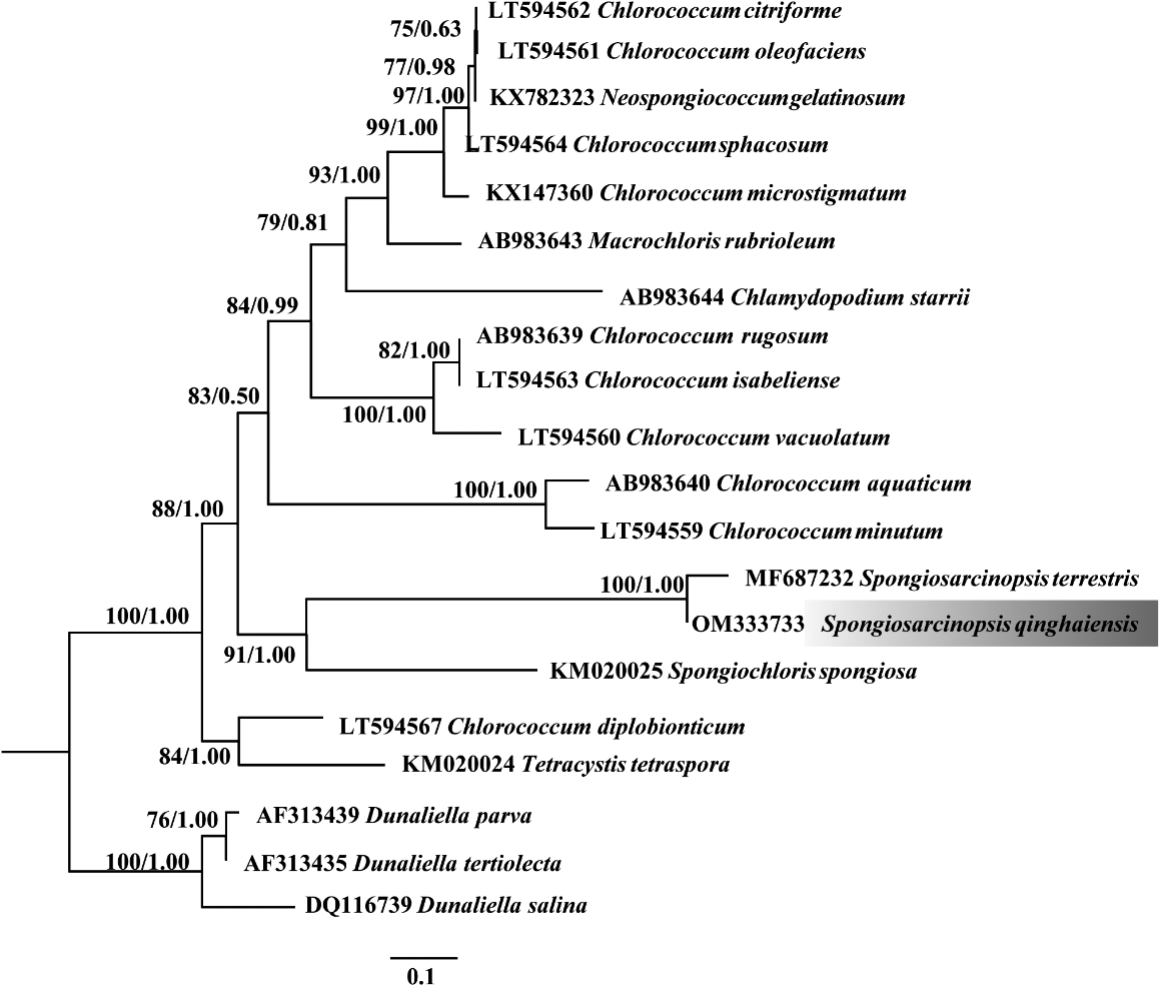
Phylogenetic tree constructed by Bayesian approach based on ITS sequences. Maximum likelihood bootstrap values and Bayesian posterior probabilities are given on nodes. Values above 0.50 for BI and 50 for ML are given. The gray part shows the new species of this study.

The phylogenetic tree was constructed using the Bayesian approach based on 18S rDNA and ITS alignments (Figs 11, 12), with the Bayesian posterior probabilities and ML bootstrap support reported. The topology of our 18S rDNA phylogeny (Fig. 11) is essentially consistent with that reported in previous studies (Temraleeva et al. 2017). The tree based on 18S rDNA showed a strong *Spongiosarcinopsis* clade, comprising the algal strains isolated in the present study; hence, the algal isolates were classified into the genus *Spongiosarcinopsis* (Fig. 11). The tree based on ITS sequences prsesented the interspecific relationships within the genus *Spongiosarcinopsis*, and the algal strain isolated in the present study formed a distinct branch separate from *Spongiosarcinopsisterrestris* (Fig. 12).

##### ﻿ITS2 secondary structure

**Figure 13.**
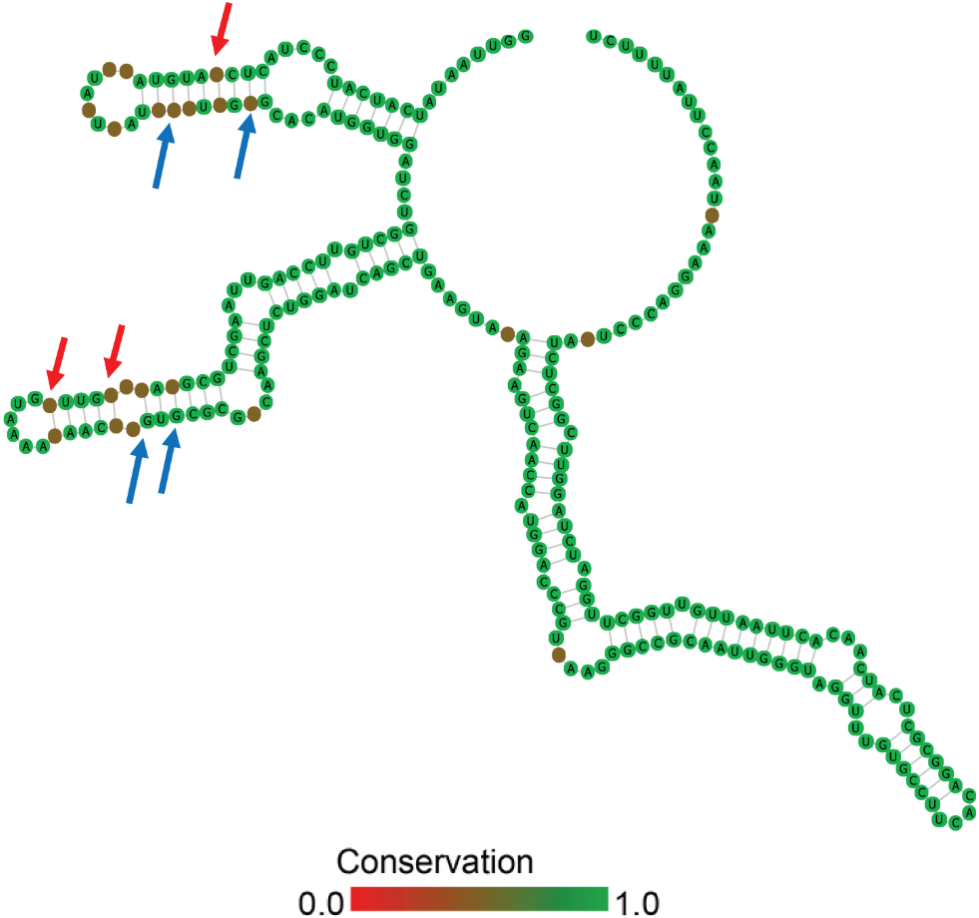
The ITS2 secondary structure between the two strains in *Spongiosarcinopsis*. The darker the color, the more significant the variation. Differences between the two species have been indicated by arrows in the figure, red for CBCs and blue for h-CBCs.

The ITS2 secondary structure was annotated, and two *Spongiosarcinopsis* strains were detected (Fig. 13), namely, *Spongiosarcinopsisterrestris* (MF687232) and *Spongiosarcinopsisqinghaiensis* (OM333733). The differences between the two strains of algae were compared. Three CBCs and four hemi-compensatory base changes (h-CBCs) were detected between the two strains.

## ﻿Discussion

Protosiphonaceae used to include *Chlorochytrium* (Cohn 1872) and *Kentrosphaera* (Borzi 1883; Kostikov 2001); however, according to algaebase records, there are only three genera left in Protosiphonaceae, namely: *Spongiosarcinopsis* (Temraleeva et al. 2018; Temraleeva 2019), *Protosiphon* (Klebs 1896), and *Urnella* (Playfair 1918). Nakada et al. (2008) includes Protoiphonaceae in Stephanosphaerinia clade, but does not take into account morphological features. Recently, Protoiphonaceae (Chlorophyta, Chlorophyceae) have been reported to be characterized by naked zoospores, one to several pyrenoids, and a starch envelope; furthermore, most algae in the family can produce excessive secondary carotenoids, so that the algae turn orange (Temraleeva et al. 2017).

In the present study, a phylogenetic tree constructed using the Bayesian approach based on 18S rDNA revealed that the algal isolates formed a robust branch and were closely related to *Spongiosarcinopsisterrestris* (MF687231) (Fig. 11). Furthermore, intraspecific, interspecific, and adjacent genera (Protosiphonaceae) showed high ML support and Bayesian posterior probability. As a boundary, *Spongiosarcinopsis* is clearly separated from the adjacent genus to form two distinct clades. Such molecular phylogenetic data support the classification of our novel algal isolate into the genus *Spongiosarcinopsis*. The phylogenetic tree constructed using the Bayesian approach based on ITS sequence showed that *S.terrestris* (MF687232) and *S.qinghaiensis* (OM333733) formed a clade but are clearly separated, and had high ML support and Bayesian posterior probability (Fig. 12). The finding was confirmed by the evolutionary tree developed based on ITS2 secondary structure (Fig. 13).

Three CBCs and four h-CBCs were detected between *S.terrestris* (MF687232) and *S.qinghaiensis* (OM333733) based on ITS2 secondary structure analysis. Analysis of the four main spiral branches revealed that the two strains had varying characteristics to different degrees. The presence of CBCs is often used as an indicator of isolated species or genera (Vanormelingen et al. 2007; Bock et al. 2011a, b). The difference suggests that *S.qinghaiensis* (OM333733) is unique.

Morphologically, unlike *S.terrestris*, the isolated strain was characterized based on habitat, young vegetative cell shape, size of vegetative cells, and number of pyrenoids. The thylakoid bundle shape is straight; however, this may not be its true state, and is not used as a distinguishing feature here. Both algal strains were found on the soil surface; however, the soil types were different. *S.qinghaiensis* was found in loose soil around a brackish lake, whereas *S.terrestris* was found in a gray forest soil. Such gaps in geography and habitat may be some of the reasons for the unique *S.qinghaiensis* morphology and phylogeny. To better distinguish *S.qinghaiensis* and *S.terrestris*, their morphological characteristics were compared. The algal strain isolated in the present study is spherical at the young stage, whereas *S.terrestris* is ellipsoidal. Furthermore, *S.qinghaiensis* has one pyrenoid and a cell diameter of 6–15 μm. In contrast, *S.terrestris* has 1–5 pyrenoids and a cell diameter of 5–10 μm. The *Spongiosarcinopsis* strain isolated in the present study was derived from the soil surface next to a brackish lake. The extreme environments with a high altitude (3201.8 m), high salinity, and low temperature suggest that there may still be numerous undiscovered algal species growing under similar environments. The molecular data and morphological characteristics support *Spongiosarcinopsisqinghaiensis* as a novel species.

## ﻿Conclusions

Based on phylogenetic analysis, ITS2 secondary structure comparison, morphological characteristics, and ultrastructure, *Spongiosarcinopsisqinghaiensis* is proposed as a novel *Spongiosarcinopsis* sp. Algal strains in highland areas are often not readily observed, which may lead to algal diversity being partially overlooked, so that more research should be undertaken.

## Supplementary Material

XML Treatment for Spongiosarcinopsis qinghaiensis
